# PRMT6-CDC20 facilitates glioblastoma progression via the degradation of CDKN1B

**DOI:** 10.1038/s41388-023-02624-7

**Published:** 2023-02-15

**Authors:** Ji Wang, Zongyu Xiao, Peng Li, Chunwang Wu, Yan Li, Qing Wang, Yanming Chen, Honglong Zhou, Zhi Li, Zhaotao Wang, Qing Lan, Yezhong Wang

**Affiliations:** 1 Department of Neurosurgery, Institute of Neuroscience, The Second Affiliated Hospital of Guangzhou Medical University, Guangzhou, 510260 China; 2 Department of Neurosurgery, Dushu Lake Hospital Affiliated to Soochow University, Suzhou, 215124 China; 3 Department of Neurosurgery, Institute of Brain Diseases, Nanfang Hospital of Southern Medical University, Guangzhou, 510515 China; 4 Department of Human and Molecular Genetics, Institute of Molecular Medicine, Massey Cancer Center, Virginia Commonwealth University, School of Medicine, Richmond, VA 23298 USA; 5 Department of Neurosurgery, The Second Affiliated Hospital of Soochow University, Suzhou, 215004 China; 6 Department of Cardiology, The First Affiliated Hospital of University of Science and Technology of China, Hefei, 230001 China; 7 Department of Neurosurgery, The Second Affiliated Hospital of Nanchang University, Nanchang, 330006 China

**Keywords:** Cancer epidemiology, CNS cancer

## Abstract

PRMT6, a type I arginine methyltransferase, di-methylates the arginine residues of both histones and non-histones asymmetrically. Increasing evidence indicates that PRMT6 plays a tumor mediator involved in human malignancies. Here, we aim to uncover the essential role and underlying mechanisms of PRMT6 in promoting glioblastoma (GBM) proliferation. Investigation of PRMT6 expression in glioma tissues demonstrated that PRMT6 is overexpressed, and elevated expression of PRMT6 is negatively correlated with poor prognosis in glioma/GBM patients. Silencing PRMT6 inhibited GBM cell proliferation and induced cell cycle arrest at the G0/G1 phase, while overexpressing PRMT6 had opposite results. Further, we found that PRMT6 attenuates the protein stability of CDKN1B by promoting its degradation. Subsequent mechanistic investigations showed that PRMT6 maintains the transcription of CDC20 by activating histone methylation mark (H3R2me2a), and CDC20 interacts with and destabilizes CDKN1B. Rescue experimental results confirmed that PRMT6 promotes the ubiquitinated degradation of CDKN1B and cell proliferation via CDC20. We also verified that the PRMT6 inhibitor (EPZ020411) could attenuate the proliferative effect of GBM cells. Our findings illustrate that PRMT6, an epigenetic mediator, promotes CDC20 transcription via H3R2me2a to mediate the degradation of CDKN1B to facilitate GBM progression. Targeting PRMT6-CDC20-CDKN1B axis might be a promising therapeutic strategy for GBM.

## Introduction

Glioblastoma (GBM) is the most frequent and destructive primary malignant brain tumor [1]. Even with current highly aggressive therapies for GBM, the median survival time of GBM patients is only 14.6 months [2]. GBMs are characterized by rapid growth and high mitotic activity, suggesting growth division mediators are the rationale for the treatment of GBM [3]. Over the past decade, clinical trials targeting key genes (such as *EGFR*, *EGFRvIII*, *CDKs*) that promote the malignant proliferation of GBM have all ended in failure, which has to make us think about novel strategies for treating GBM [4, 5]. Growing evidence suggests that various epigenetics, such as aberrant DNA or RNA methylation, and changes in the status of protein modification, might play an important role in GBM initiation and progression [6, 7]. For example, more than 50% of GBMs contain homozygous p16INK4a gene deletion by hypermethylation, and restoration of p16INK4a inhibits cell proliferation and induces cell cycle arrest [8]. m6A reader YTHDF2 activates the NF-κB pathway to promote the malignant progression of GBM [9]. Recent investigations have found that protein modification enzymes such as HDACs and EZH2 control the methylation level of target proteins to regulate gene transcription in GBM [10, 11]. Progress in this field suggests that these epigenetic alterations will be commonly used shortly to direct the prevention and treatment of GBM [6].

Arginine methylation is a prevalent post-translational modification catalyzed by protein arginine methyltransferases (PRMTs) [12]. PRMTs catalyze mono- or di-methylation of arginine residues. According to different catalytic specificities, asymmetric dimethylarginine is generated by type I PRMTs (PRMT1-4, PRMT6, and PRMT8), while symmetric dimethylarginine forms with the help of type II PRMTs (PRMT5 and PRMT9) [13]. Current protein methylomes indicate that almost 3% of metabolic enzymes are modified by arginine methylation [14]. Protein arginine methylation is responsible for the regulation of cell metabolism, transcription, mRNA translation, and signal transduction [15]. PRMT6, a type I PRMT, di-methylates the arginine residues of both histones and non-histones asymmetrically [16]. Numerous pieces of evidence suggest that PRMT6 is involved in a variety of cellular functions, including cellular division, RNA metabolism, cellular senescence, and cellular homeostasis [17]. Now, it is widely reported that PRMT6 acts as a tumor mediator and exerts a dual role in human cancer. In hepatocellular carcinoma, there is a significant correlation between decreased PRMT6 expression and short overall survival, whereas PRMT6 overexpression is associated with tumorigenicity and invasiveness in gastric cancer and lung cancer [17, 18]. It has been described as an arginine methyltransferase that methylates CRAF, P16, and BAG5 proteins involved in tumor progression [19–21]. In the present study, Huang et al. showed that PRMT6 methylation of RCC1 is responsible for the regulation of mitotic, tumorigenicity, and radiation-induced response of glioblastoma stem cells, suggesting PRMT6 may act as an oncogene involved in glioma [22]. Nonetheless, the role of PRMT6 in carcinogenesis and progression of GBM remains poorly understood.

Cell division cycle 20 (CDC20) is a well-known regulator of the cell cycle, which binds with the Anaphase Promoting Complex (APC) to form E3 ubiquitin ligase sub-complexes, and plays a pivotal role in the segregation of chromosomes during mitosis [23]. The accumulation of evidence has demonstrated that CDC20 is dramatically elevated in human malignancies (including pancreatic cancer, breast cancer, and lung cancer), and CDC20 contributes to the malignant progression of cancer by degradation of its downstream target genes (such as CDKN1A (P21), Cyclin B1, and Bim) through ubiquitination, making it a promising target for cancer treatment [24]. In glioma, CDC20 has been identified as an oncogenic factor mediating tumor cell growth, invasion, and chemosensitivity [25]. Targeting CDC20 inhibits the malignant progression of glioma and CDC20 is considered an attractive target for therapeutic intervention [26, 27]. Larsen et al. performed a proteome-wide analysis of arginine monomethylation in human cells and discovered that there are several cell cycle–associated protein complexes rich in arginine methylation, including MAD2L1, BUB3, and CDC20 [28]. This finding indicates that PRMTs might provide a novel insight into CDC20 regulation. However, it is not clear how PRMT6 interacts with CDC20 in GBM.

In this study, we report that PRMT6 contributes to GBM cell proliferation in vitro and in vivo. Mechanical research has shown that PRMT6 epigenetically facilitates the transcription of the *CDC20* gene via H3R2me2a, thereby promoting the ubiquitination and degradation of CDKN1B (P27). Further analysis showed that PRMT6 small molecule inhibitor (EPZ020411) intervention exerted an inhibitory effect on GBM cell proliferation in vitro. Our results suggest that the PRMT6-CDC20 axis may serve as a prognostic and therapeutic target for GBM.

## Results

### Elevated *PRMT6* expression in gliomas correlates with poor patient prognosis

**Fig. 1:**
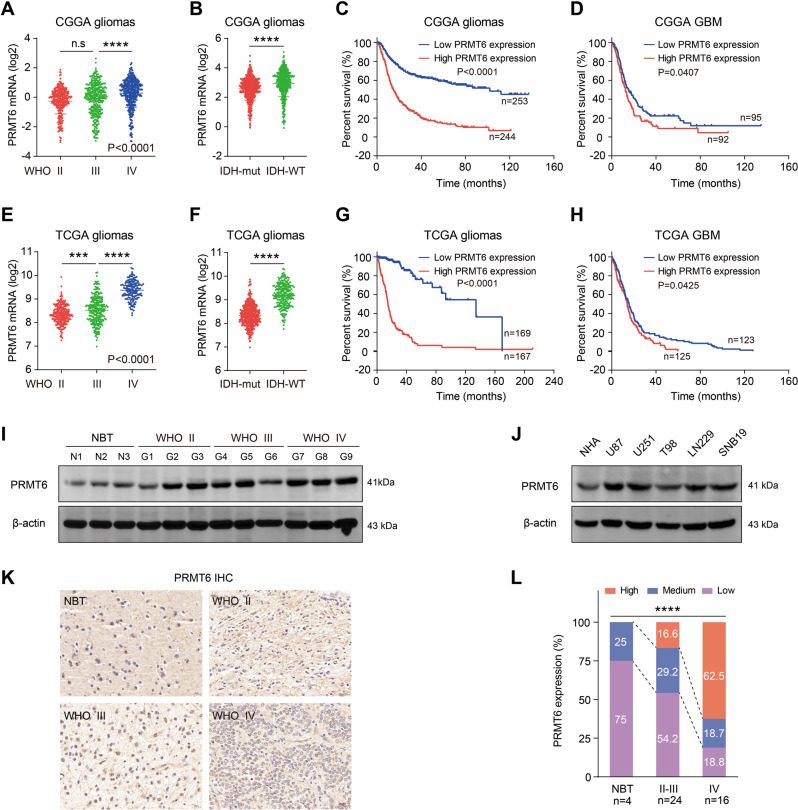
PRMT6 is overexpressed in glioma. **A**, **B** PRMT6 mRNA expression in gliomas with different WHO grades and different IDH statuses from the CGGA database. **C**, **D** Kaplan-Meier survival curves of patients with glioma and GBM from the CGGA database stratified by PRMT6 expression. **E**, **F** PRMT6 mRNA expression in gliomas with different WHO grades and different IDH statuses from the TCGA database. **G**, **H** Kaplan-Meier survival curves of patients with glioma and GBM from the TCGA database stratified by PRMT6 expression. **I** The protein expression of PRMT6 in fresh glioma tissues was measured by western blotting. **J** The protein expression of PRMT6 in different glioblastoma cell lines and NHA were detected by western blotting. **K** Representative IHC images of PRMT6 in gliomas with different histological grades and NBT. Bar: 20 µm. **L** The semi‑quantitative for the IHC results of PRMT6. n.s: no significant, ***p < 0.001, ****p < 0.0001.

To understand the expression profiles of *PRMT6* in glioma tissues and their prognostic significance, we analyzed the sequencing data of glioma in CGGA, TCGA, and Gravendeel databases. Here, we confirmed an elevated *PRMT6* expression in higher WHO grades gliomas (Fig. 1A, E) and in IDH-wild-type gliomas (Fig. 1B, F). Then, we investigated the prognostic significance of *PRMT6* expression in patients with glioma, and the results showed that patients with high levels of *PRMT6* had poorer overall survival compared to those with low *PRMT6* expression (Fig. 1C, G). In addition, the overall survival rate of GBM patients could also be determined by *PRMT6* expression (Fig. 1D, H). We also observed similar results in Gravendeel dataset (Fig. S1A–D). Furthermore, the results of immunoblotting on the freshly collected protein samples from gliomas revealed markedly higher PRMT6 protein levels compared to NBT, particularly in Grade III and IV gliomas (Fig. 1I). The PRMT6 protein levels in the majority of glioblastoma cells (U87, U251, LN229, SNB19) were also higher than that of NHA (Fig. 1J). Immunohistochemistry (IHC) images also showed that PRMT6 was increased with increasing WHO grades in glioma, and higher than NBT (Fig. 1K, L). Taken together, these results suggest that the increase in PRMT6 might be associated with oncogenesis in glioma.

### PRMT6 induces GBM cell proliferation

**Fig. 2:**
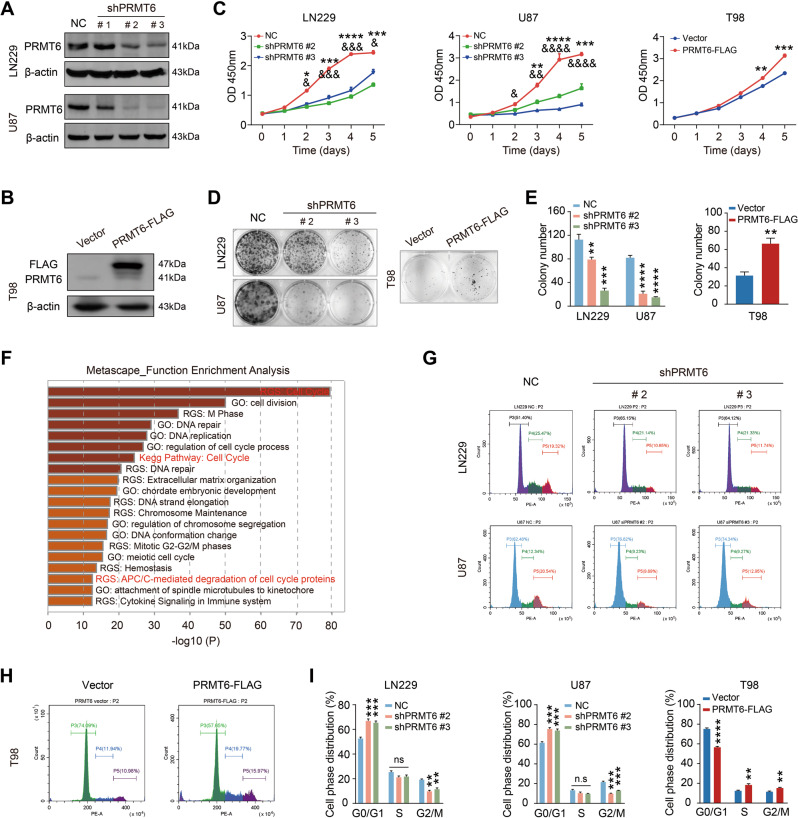
PRMT6 promotes GBM cell proliferation. **A**, **B** The protein expression of PRMT6 was detected in LN229 and U87 cells transfected with PRMT6 shRNA or NC shRNA lentivirus and T98 cells transfected with PRMT6 overexpression or vector plasmids by immunoblotting. **C** Cell proliferation was evaluated in LN229 and U87 cells with or without PRMT6 depletion and T98 cells with or without PRMT6 overexpression by CCK-8 assay. LN229, U87 cells: PRMT6 shRNA #2 versus NC shRNA, *P < 0.05, ***P < 0.001, ****P < 0.0001; PRMT6 shRNA #3 versus NC shRNA, &*P* < 0.05, &&*P* < 0.01, &&&*P* < 0.001, &&&&*P* < 0.0001. **D** Colony formation capability was examined in LN229 and U87 cells with or without PRMT6 depletion and T98 cells with or without PRMT6 overexpression by colony-formation assay. **E** Quantifications of colony-formation assay results. **F** Functional enrichment analysis terms of the 1183 genes whose expression is positively correlated with PRMT6 expression in GBM from the TCGA database. **G**, **H** Cell cycle analysis of LN229 and U87 cells with or without PRMT6 inhibition and T98 cells with or without PRMT6 overexpression was performed by flow cytometry. **I** Quantifications of cell cycle analysis results. n.s: no significant, **p < 0.01, ***p < 0.001, ****p < 0.0001.

To investigate the effect of PRMT6 expression on GBM cells, we constructed PRMT6-silenced or PRMT6-overexpressed cell models by PRMT6 shRNA lentivirus or PRMT6 ORF plasmid transfection. Immunoblotting results revealed that PRMT6 protein expression was significantly suppressed in LN229, and U87 cells, and was overexpressed in T98 cells (Fig. 2A, B). Then, we performed CCK-8 assays and found that silencing PRMT6 significantly attenuated the proliferation of LN229, and U87 cells, and overexpression of PRMT6 promoted the growth of T98 cells (Fig. 2C). Colony formation assays were also carried out to evaluate the proliferative effect of PRMT6 on GBM cells. The GBM cell colony numbers demonstrated that the expression of PRMT6 strongly induced GBM cell proliferation (Fig. 2D, E). To further investigate the potential role of PRMT6 on GBM, we identified the genes that positively correlated with PRMT6 expression in GBM in the TCGA database. Functional enrichment analysis results showed that these genes were associated with malignancy-related biological processes, such as cell cycle and DNA replication (Fig. 2F). Cell cycle is accompanied by cell proliferation and is mainly the biological process of cell growth. Thus, we explored the influence of PRMT6 on the cell cycle distribution of GBM cells. Flow cytometry results showed that PRMT6 exacerbated the G1/S phase transition of GBM cells (Fig. 2G–I). Together, these data demonstrate that elevated expression of PRMT6 could induce GBM cell proliferation.

### PRMT6 attenuates the protein stability of CDKN1B by promoting its degradation

**Fig. 3:**
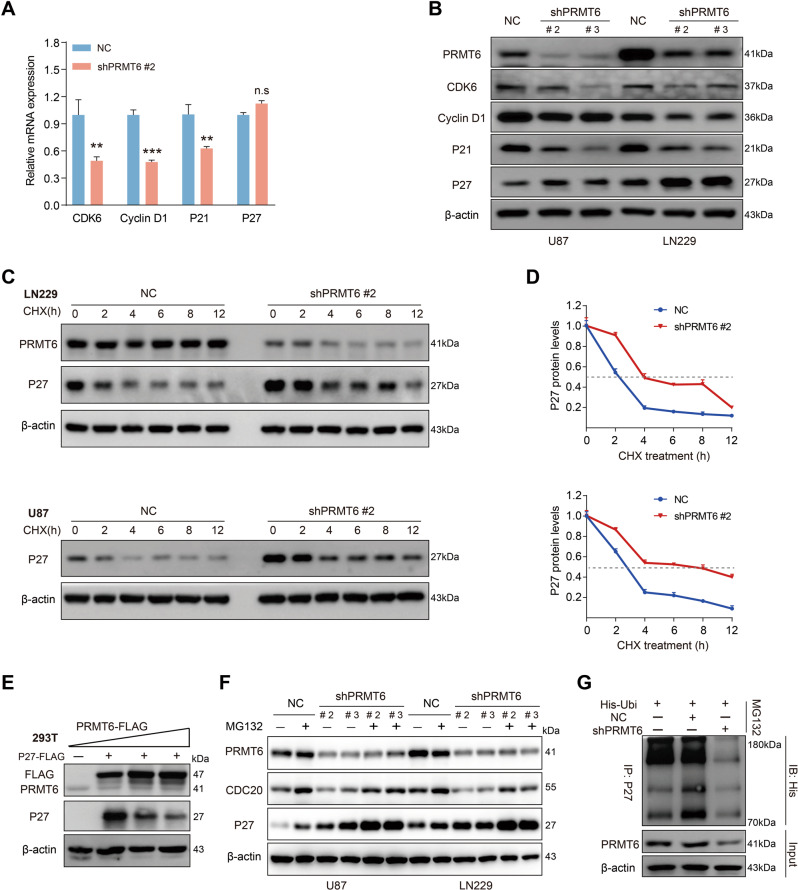
PRMT6 attenuates the protein stability of CDKN1B by the ubiquitin-proteasome pathway. **A** RT-PCR analysis evaluated the mRNA expression of CDK6, Cyclin D1, CDKN1A (P21), and CDKN1B (P27) in U87 NC or U87 shPRMT6 #2 cells. **B** The immunoblotting analysis examined the protein expression of PRMT6, CDK6, Cyclin D1, CDKN1A, and CDKN1B in U87 and LN229 cells with or without PRMT6 depletion. **C** Immunoblotting analysis of LN229 and U87 cells with or without PRMT6 knockdown to measure the protein half-life of CDKN1B. **D** Quantifications of the protein half-life results. **E** The protein expression of PRMT6 and CDKN1B were evaluated in HEK293T cells transfected with CDKN1B-FLAG and different doses of PRMT6-FLAG plasmids by immunoblotting. **F** U87 and LN229 cells, with or without PRMT6 depletion, were treated with vehicle control or with MG132 (20 μM) for 6 h, and the abundance of CDKN1B was determined by western blotting. **G** U87 cells with or without PRMT6 depletion were transfected with His-Ubi and then were treated with MG132 (20 μM) for 6 h. Cell lysates were immunoprecipitated with an anti-CDKN1B antibody and CDKN1B ubiquitination was tested by western blotting. n.s: no significant, **p < 0.01, ***p < 0.001.

To elucidate how PRMT6 enhances the G1/S transition in GBM cells, we examined the effect of PRMT6 silencing on the expression of key cell cycle molecules in the G0/G1 phase. qRT-PCR results showed that CDK6, Cyclin D1, and CDKN1A mRNA levels were downregulated in PRMT6-knockdown U87 cells, while CDKN1B mRNA levels had no significant difference compared with the control cells (Fig. 3A). Then, western blotting analysis found that CDK6, Cyclin D1, and CDKN1A protein expression were inhibited in PRMT6-silenced GBM cells. Interestingly, the protein expression of CDKN1B was significantly upregulated with the inhibition of PRMT6 expression in GBM cells (Fig. 3B), implying that PRMT6 may regulate the expression of CDKN1B by post-translational modification. Studies confirm that CDKN1B is one of the common cell cycle regulatory molecules degraded by the ubiquitinated proteasome [29, 30]. To determine whether PRMT6 regulates CDKN1B protein stability, we measured the abundance of CDKN1B in PRMT6 depletion GBM cells and the control cells treated with CHX. Immunoblotting results showed that CDKN1B was greatly stabilized in LN229 or U87 cells with the absence of PRMT6 (Fig. 3C, D), while the protein half-life of CDKN1B was significantly shortened in T98 cells with the abundance of PRMT6 (Fig. S2A, B). Then, to explore the protein expression correlation of PRMT6 and CDKN1B, we transfected PRMT6 overexpression plasmids in a dose-dependent manner into HEK293T cells and found that the protein expression of CDKN1B gradually decreased (Fig. 3E). We investigated whether PRMT6 mediates the protein stability of CDKN1B by the proteasome system. A proteasome inhibitor (MG132) was used to treat the GBM cells with PRMT6 knockdown and found that the protein expression of CDKN1B was further upregulated (Fig. 3F). In T98 cells with PRMT6 overexpression, the protein of CDKN1B was re-expressed with MG132 treatment (Fig. S2C). Furthermore, we evaluated the effect of PRMT6 on CDKN1B ubiquitination, and the result showed that depletion of endogenous PRMT6 by shRNA decreased CDKN1B ubiquitination in U87 cells (Fig. 3G). Thus, these results indicate that PRMT6 attenuates the protein stability of CDKN1B by promoting its ubiquitinated degradation.

### PRMT6 is essential in maintaining the transcription of CDC20 in GBM cells

**Fig. 4:**
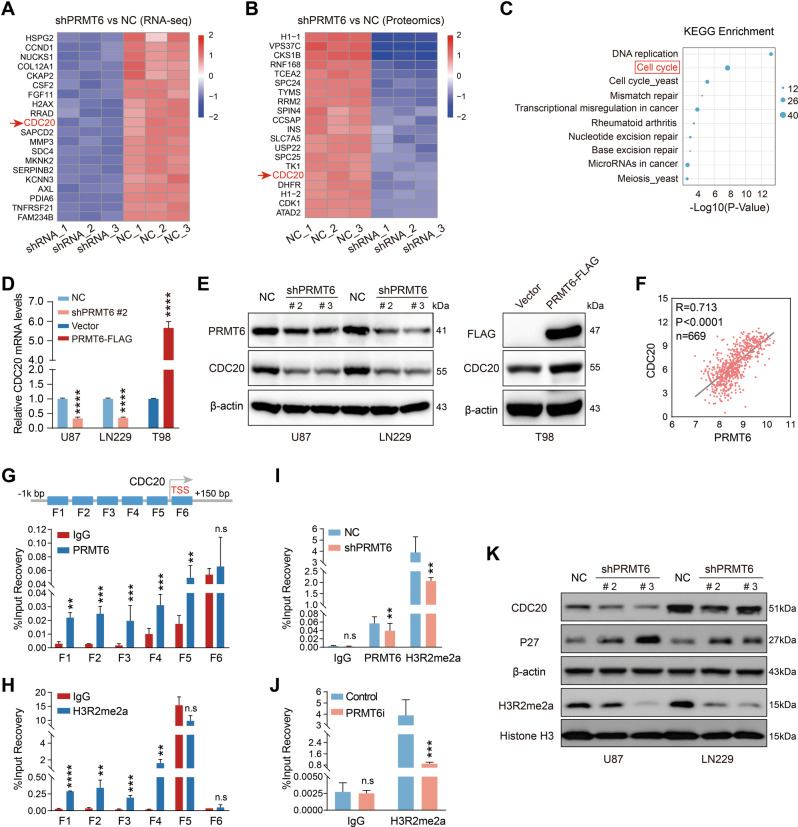
PRMT6 promotes the transcription of CDC20 in GBM cells by H3R2me2a. **A** The heatmap of RNA-seq displays the top 20 down-regulated genes in PRMT6-knockdown U87 cells. **B** The heatmap of proteomics shows the top 20 downregulated genes in PRMT6-knockdown U87 cells. **C** Bubble chart that illustrates the top 10 signaling pathways enriched based on the KEGG pathway analysis of proteomics. **D** RT-PCR analysis evaluated the mRNA expression of CDC20 in LN229 and U87 cells transfected with PRMT6 shRNA or NC shRNA lentivirus and T98 cells transfected with PRMT6 overexpression or vector plasmids. **E** The immunoblotting analysis detected the protein expression of CDC20 in U87 and LN229 cells with or without PRMT6 depletion and T98 cells with or without PRMT6 overexpression. **F** The expression correlation between PRMT6 and CDC20 of patients with GBM from the TCGA dataset. **G**, **H** Schematics depicting the detailed information of the potential PRMT6 or H3R2me2a-binding sites on the CDC20 promoter are shown, and ChIP-qPCR analysis was performed to detect PRMT6 and H3R2me2a in the promoter region of CDC20 (+150 bp~−1000bp). IgG was used as a negative control. **I, J** ChIP-qPCR was performed to determine H3R2me2a in the promoter of CDC20 (F3) in U87 cells with or without PRMT6 knockdown (shPRMT6) and U87 cells with or without PRMT6 inhibition (PRMT6i, 20 μM EPZ020411 treatment). **K** The immunoblotting analysis measured the protein expression of CDC20, CDKN1B, and H3R2me2a in U87 and LN229 cells with or without PRMT6 depletion. n.s: no significant, *p < 0.05, **p < 0.01, ***p < 0.001, ****p < 0.0001.

To further unveil the molecular mechanism of PRMT6 regulation of CDKN1B, we performed RNA sequencing and proteome-wide analysis in shPRMT6 U87 cells and the control cells. Heatmap displayed the top 20 most significantly downregulated molecules at RNA and protein levels in U87 cells with PRMT6 knockdown, respectively (Fig. 4A, B). Notably, both mRNA and protein levels of CDC20 were significantly down-regulated with PRMT6 expression inhibition. Then, KEGG enrichment analysis for differential proteins of proteomic result revealed that DNA replication and cell cycle were significantly enriched (Fig. 4C). This result was consistent with previous findings. qRT-PCR and immunoblotting analysis examined the effect of PRMT6 on CDC20 expression, and found that depletion of PRMT6 inhibited the mRNA and protein levels of CDC20, whereas PRMT6 overexpression promoted the expression of CDC20 in GBM cells (Fig. 4D, E). Moreover, we analyzed the correlation between PRMT6 and CDC20 expression in GBM patients from the TCGA dataset and showed that the expression of PRMT6 and CDC20 were strongly correlated (Fig. 4F). Bioinformatics analysis of TCGA and CGGA datasets results showed that CDC20 was overexpressed in glioma, especially in GBM, and the increasing of CDC20 in glioma patients correlated with poorer prognosis (Fig. S3A-D). In addition, IHC images of the collected glioma samples also demonstrated that CDC20 were increased with the rise of WHO grades in glioma, and higher than NBT (Fig. S3E, F). Based on IHC staining results, we analyze the clinical relevance of PRMT6 to CDC20, and the data showed that expression levels of PRMT6 were closely correlated with those of CDC20 (Fig. S3G). Integrating RNA sequencing, proteome-wide analysis, RT-qPCR, immunoblot and IHC results, PRMT6 positively regulates the transcription of the *CDC20* gene in GBM cells. PRMT6 can activate gene transcription by modulating histone methylation, most notably asymmetric di-methylation on arginine 2 of histone 3 (H3R2me2a) [31]. Thus, we examined PRMT6 and H3R2me2a in the 150 bp∼−1kbp region spanning the promoter of CDC20 in GBM cells and revealed positive enrichment for PRMT6 and H3R2me2a, in the 1000 bp∼200 bp upstream of the CDC20 transcriptional start site (Primer F1-F4) (Fig. 4G, H and Fig. S3H, I). Independent ChIP-qPCR assays verified the lower occupancy of H3R2me2a at the loci of CDC20 promoter (Primer F3 and F4) in U87 cells with PRMT6 silencing or inhibition (PRMT6 inhibitor, EPZ020411) (Fig. 4I, J and Fig. S3J-M). Furthermore, immunoblotting data confirmed that the knockdown of PRMT6 led to a global reduction in H3R2me2a and CDC20 (Fig. 4K). Finally, these results uncovered an essential role of PRMT6 in sustaining CDC20 expression: PRMT6 accomplishes this by adding activating histone methylation mark (H3R2me2a).

### The PRMT6-CDC20 axis maintains the proteostasis of CDKN1B

**Fig. 5:**
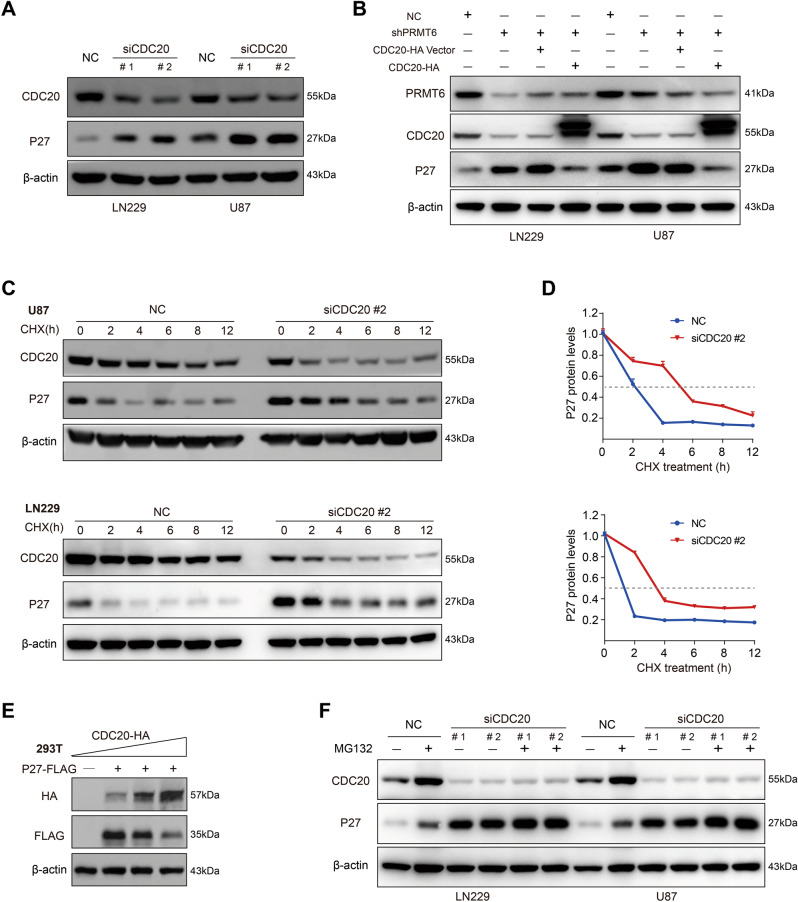
The PRMT6-CDC20 axis maintains the proteostasis of CDKN1B. **A** The protein expression of CDC20 and CDKN1B in U87 and LN229 cells transfected with CDC20 siRNAs or NC were evaluated by western blotting. **B** Immunoblot analysis of CDC20 and CDKN1B expression in U87 and LN229 cells treated with NC shRNA, shPRMT6, shPRMT6 + CDC20 Vector, or shPRMT6 + CDC20. **C** Immunoblotting analysis of LN229 and U87 cells with or without CDC20 knockdown to examine the protein half-life of CDKN1B. **D** Quantifications of the protein half-life results. **E** The protein expression of CDC20 (HA) and CDKN1B (FLAG) were detected in HEK293T cells transfected with CDKN1B-FLAG and different doses of CDC20-HA plasmids by western blotting. **F** U87 and LN229 cells, with or without CDC20 knockdown, were treated with vehicle control or with MG132 (20 μM) for 6 h, and the abundance of CDKN1B was determined by immunoblotting.

CDC20 is a regulatory protein, that interacts with several other proteins at multiple points in the cell cycle [32]. CDC20 forms the CDC20-APC/C complex with APC/C, which subsequently degrades its substrates through the ubiquitination system, including CDKN1A, Cyclin B1, and Cyclin A2 [33–35]. Downregulated CDC20 expression and higher abundance of the CDKN1B protein in PRMT6 deficient cells led us to hypothesize that PRMT6-CDC20 axis regulates CDKN1B proteostasis. First, immunoblotting results revealed that the inhibition of CDC20 resulted in a higher level of CDKN1B (Fig. 5A). Then, we transfected the CDC20 overexpression plasmid into PRMT6-silenced GBM cells to achieve CDC20 re-expression, and found that the expression of CDKN1B was downregulated (Fig. 5B). This effect indicates that PRMT6 reduced the protein stability of CDKN1B mediated by CDC20 in GBM cells. To evaluate the effect of CDC20 on CDKN1B protein stability, we examined the abundance of CDKN1B in CDC20 depletion or overexpression GBM cells and the control cells treated with CHX. The data revealed that CDKN1B was greatly stabilized in LN229 or U87 cells with the absence of CDC20 (Fig. 5C, D), while the protein half-life of CDKN1B was significantly shortened in T98 cells with the abundance of CDC20 (Fig. S4A, B). The protein expression correlation of CDC20 and CDKN1B in HEK293T demonstrated that CDKN1B was negatively correlated with the expression of CDC20 (Fig. 5E). MG132 was used to treat the GBM cells with CDC20 knockdown and found that the protein expression of CDKN1B was further upregulated (Fig. 5F). In T98 cells with CDC20 overexpression, the protein of CDKN1B was re-expressed with MG132 treatment (Fig. S4C). These results suggest that PRMT6 maintains CDC20-mediated CDKN1B proteostasis in GBM cells.

### CDC20 interacts with and destabilizes CDKN1B

**Fig. 6:**
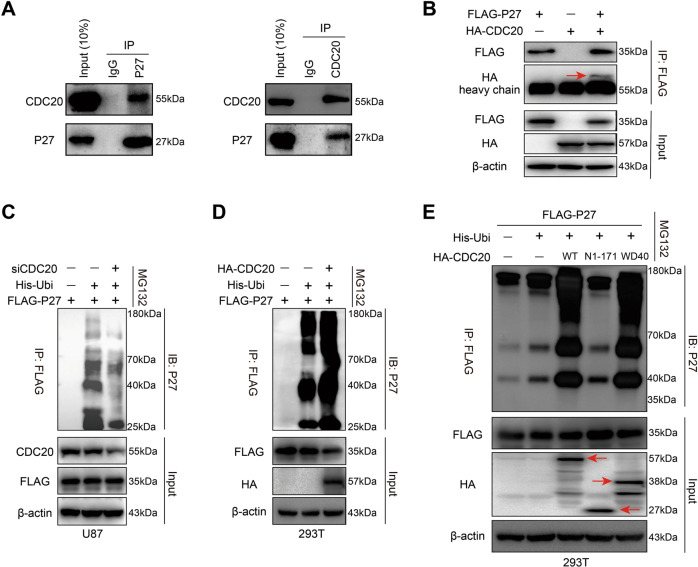
CDC20 interacts with and destabilizes CDKN1B. **A** The interaction of endogenous CDC20 with CDKN1B was evaluated by Co-IP assay in U87 cells. **B** The interaction of exogenous CDC20 with CDKN1B was assessed by Co-IP assay in HEK293T cells. **C** U87 cells with or without CDC20 depletion were transfected with His-Ubi and CDKN1B-FLAG plasmids and then were treated with MG132 (20 μM) for 6 h. Cell lysates were immunoprecipitated with an anti-FLAG antibody and CDKN1B ubiquitination was detected by immunoblotting. **D** HEK293T cells with or without CDC20 overexpression were transfected with His-Ubi and CDKN1B-FLAG plasmids and then were treated with MG132 (20 μM) for 6 h. Cell lysates were immunoprecipitated with an anti-FLAG antibody and CDKN1B ubiquitination was examined by immunoblotting. **E** HEK293T cells were transfected with CDC20-HA WT or CDC20-HA-N1-171 or CDC20-HA-WD40, CDKN1B-FLAG, and His-Ubi expressing plasmids, and then were treated with MG132 (20 μM) for 6 h. Cell lysates were immunoprecipitated with an anti-FLAG antibody and CDKN1B ubiquitination was determined by immunoblotting.

To identify CDC20 responsible for the negative proteostasis of CDKN1B, Co-IP experiments were conducted in U87, LN229, and HEK293T cells. We found that both endogenous and exogenous CDKN1B interact with CDC20 (Fig. 6A, B). In Fig. S5A, we observed that both endogenous CDKN1B and CDC20 were mainly co-localized in the nucleus in U87 and LN229 cells. Moreover, we wondered whether CDC20 can directly mediate CDKN1B ubiquitination. We performed an in vivo ubiquitination assay by co-transfection of CDKN1B-FLAG, Ubiquitin-His, and CDC20 siRNA or CDC20-HA plasmids. The results showed that ubiquitination of CDKN1B by CDC20 was attenuated with CDC20 silencing in U87 cells, while ubiquitination of CDKN1B by CDC20 was enhanced with CDC20 overexpression in HEK293T cells (Fig. 6C, D). Further, overexpression of a WD40 repeat domain from CDC20, an active form of CDC20 that mediates protein-protein interaction and ubiquitination [36], enhanced CDKN1B ubiquitination (Fig. 6E). These results demonstrate that CDKN1B’s binding with and ubiquitination by CDC20 is critical for PRMT6-mediated downregulation of CDKN1B.

### Inhibition of PRMT6 attenuates the proliferative effect of GBM cells

**Fig. 7:**
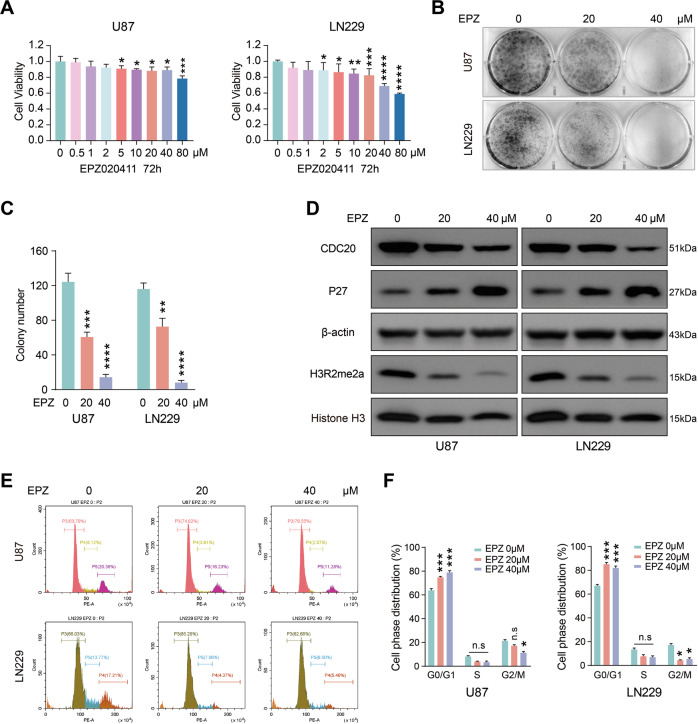
EPZ020411 suppresses GBM cell proliferation. **A** Cell viability for U87 and LN229 cells were treated with the indicated concentration of EPZ020411 for 72 h. **B** Colony formation capability for U87 and LN229 cells treated with the indicated concentration of EPZ020411. **C** Quantifications of colony-formation assay results. **D** Immunoblotting analysis for the protein expression of CDC20, CDKN1B, and H3R2me2a in U87 and LN229 cells treated with the indicated concentration of EPZ020411 for 48 h. **E** Flow cytometry analysis for cell cycle distribution in LN229 and U87 cells treated with the indicated concentration of EPZ020411 for 48 h. **F** Quantifications of cell cycle analysis results. n.s: no significant, *p < 0.05, **p < 0.01, ***p < 0.001, ****p < 0.0001.

Given that silencing of PRMT6 inhibits GBM cell proliferation by maintaining CDKN1B expression, we were trying to examine whether inhibition of PRMT6 would exert a better therapeutic effect on GBM cells. To this end, we measured the effect of the PRMT6 inhibitor (EPZ020411) on the proliferation of GBM cells. CCK-8 results showed that EPZ020411 improved the anti-proliferative effect on U87 and LN229 cells in a dose-dependent manner (Fig. 7A). Colony formation assays results also revealed that EPZ020411 could suppress the proliferation of GBM cells (Fig. 7B, C). Then, we investigated biological activity of PRMT6 protein, and immunoblotting data demonstrated that EPZ020411 decreased the levels of CDC20 and H3R2me2a, while increasing the expression of CDKN1B, indicating EPZ020411 specificity for inhibiting PRMT6 (Fig. 7D). Previous data have confirmed that PRMT6 affects cell cycle progression in GBM cells. Thus, we tested whether PRMT6 inhibitor similarly regulates the cell cycle of GBM cells. Flow cytometry results revealed that EPZ020411-treated GBM cell phase distribution was arrested at the G0/G1 phase (Fig. 7E, F). Collectively, these data indicate that targeting intervention with EPZ020411 treatment had an inhibitory effect on the proliferation of GBM cells in vitro.

### PRMT6 contributes to GBM cell proliferation via CDC20 in vitro and in vivo

**Fig. 8:**
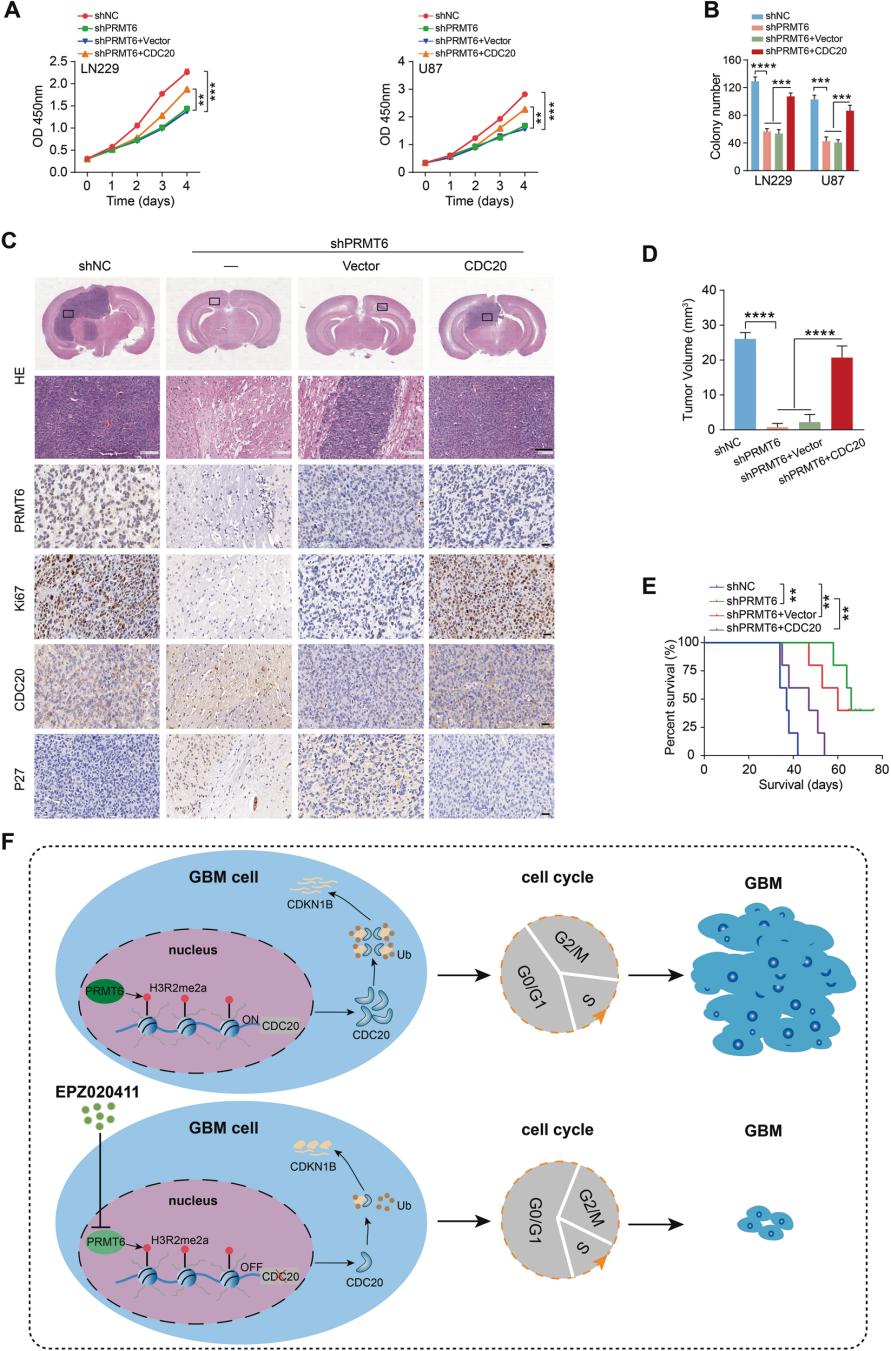
PRMT6 promotes GBM cell proliferation via CDC20 in vitro and in vivo. **A** Cell viability for U87 and LN229 cells infected with NC shRNA, shPRMT6, shPRMT6 + CDC20 Vector or shPRMT6 + CDC20. **B** Colony formation capability for U87 and LN229 cells infected with NC shRNA, shPRMT6, shPRMT6 + CDC20 Vector or shPRMT6 + CDC20. **C** Control, shPRMT6, shPRMT6 + CDC20 Vector, or shPRMT6 + CDC20-infected U87 cells were intracranially injected into nude mice, and representative HE images and indicated IHC images of xenograft tumors are shown. HE bar: 100 µm, IHC bar: 20 µm. **D** Quantification of the tumor volumes. **E** Kaplan-Meier survival curve shows the survival times of Control, shPRMT6, shPRMT6 + CDC20 Vector, or shPRMT6 + CDC20-infected U87 cells xenograft mice. **F** Schematic model of PRMT6-CDC20-CDKN1B axis-mediated cell proliferation and GBM tumorigenesis. **p < 0.01, ***p < 0.001, ****p < 0.0001.

To confirm the role of CDC20 in PRMT6 inducing GBM cell proliferation, we transfected CDC20 overexpression lentiviruses into shPRMT6 cells for rescue experiments (Fig. S6A). CCK-8 and colony formation assays revealed that the rescue of CDC20 largely restored the viability of PRMT6-silenced GBM cells (Fig. 8A, B and S6B). Additionally, we examined the effects of the PRMT6-CDC20 axis in the carcinogenesis of GBM in vivo. Xenotransplantation of U87 cells confirmed that PRMT6 knockdown markedly inhibited the growth of transplanted tumors, while CDC20 re-expression partially counteracted the aforementioned PRMT6 silencing effect (Fig. 8C, D). KM survival analysis revealed that the survival time of xenograft mice was significantly prolonged after PRMT6 inhibition, while the CDC20 rescue xenograft mice had a similar survival time to the control mice (Fig. 8E). IHC staining was used to measure the expression of related molecules in xenograft mice brains. The IHC images showed a lower positive rate of PRMT6, Ki67, CDC20, and a higher expression of CDKN1B in PRMT6-knockdown tumors. In the CDC20 rescue U87 tumors, we observed significant upregulation of Ki67, CDC20, and noticeably downregulation of CDKN1B compared to PRMT6-knockdown tumors; however, the expression of PRMT6 had no differential (Fig. 8C). These evidence confirm that PRMT6 plays a role in promoting the proliferation of GBM by regulating the expression of CDC20 in vitro and in vivo.

## Discussion

In the present study, we have identified a critical role for PRMT6 in CDKN1B degradation via enhancing CDC20-mediated ubiquitination, which contributes to GBM cell proliferation and GBM tumorigenesis (Fig. 8F). Our mechanistic and clinical findings establish that the PRMT6-CDC20-CDKN1B axis is critical for GBM tumorigenesis and targeting this axis is a potential therapeutic strategy against GBM.

PRMT6 is dramatically elevated in a variety of human malignancies, including lung [37], breast [38], prostate [39], and colon cancer [40], implying an important role for PRMT6 in tumors. In previous reports, there are relatively rare studies on PRMT6 in brain tumors. Recently, Huang et al. demonstrated that PRMT6 regulates mitosis, tumorigenicity, and radiation responses in glioblastoma stem cells by methylating RCC1 [22], which implicates PRMT6 as an important oncogene in GBM. A key finding in this study is the elucidation that PRMT6 methylates RCC1 to induce cell cycle progression to promote mitosis. However, how the PRMT6-RCC1 axis specifically regulates the cell cycle of GBM cells has not been investigated. In our study, we confirmed that PRMT6 acts as an oncogene in gliomas to facilitate GBM cell proliferation. Further, we performed a functional enrichment analysis of genes closely related to PRMT6 expression in GBM in the TCGA database and found that cell cycle was the function most closely related to PRMT6. PRMT6 regulation of the cell cycle of tumor cells has been reported in previous studies [41, 42], and we also verified cell cycle is arrested at G0/G1 phase after PRMT6 silencing in GBM cells. In addition, we examined the expression of key cell cycle regulatory molecules in the G0/G1 phase. Expectedly, the mRNA and protein levels of CDK6 and CyclinD1 were downregulated with PRMT6 inhibition. Notably, the transcription of CDKN1A was significantly repressed in PRMT6 depletion GBM cells. Luo et al. and Almeida-Rios et al. reported that PRMT6 inhibits the expression of CDKN1A via histone H3R2me2-mediated repression of transcription [39, 43]. Whereas, Nakakido et al. reported that PRMT6 could increase cytoplasmic localization of CDKN1A in cancer cells through arginine methylation to promote CDKN1A expression [44]. Whether PRMT6 can directly methylate CDKN1A to promote its cytoplasmic localization in GBM cells needs further research to confirm. Here, as a well-known G0/G1 phase cell cycle suppressor, the expression level of CDKN1A does not match the cell cycle distribution, whether PRMT6 regulates the cell cycle through other mechanisms. We found the protein level of CDKN1B was increased in PRMT6-silenced cells, whereas the mRNA level was almost unchanged, suggesting that PRMT6 inhibits the expression of CDKN1B through post-translational modification. PRMT6 has also been reported to suppress CDKN1B expression in previous studies, but whether it is through H3R2me2a-mediated transcriptional repression is controversial [39, 43, 45]. RNA-sequencing results as well as the experimental result in our study demonstrated that PRMT6 does not affect the mRNA level of CDKN1B in GBM cells, and PRMT6 attenuates the protein stability of CDKN1B by the ubiquitinated proteasome. This finding suggests that PRMT6 destabilizes CDKN1B to promote cell cycle progression of GBM cells, which is different from previous reports.

CDKN1B binds and prevents the activation of Cyclin D-CDKs complexes, thereby controlling cell cycle progression in the G1 phase. The degradation of CDKN1B is triggered by its CDK-dependent phosphorylation and subsequent ubiquitination of the SCF complex, which is required for cells to transition from a quiescent to a proliferating state [46]. Therefore, CDKN1B protein is frequently degraded as a substrate for ubiquitination to promote tumor cell proliferation [47]. Given that PRMT6 mediates the downregulation of CDKN1B protein expression via the ubiquitin-proteasome, we hypothesized that PRMT6 degrades CDKN1B via an E3 ubiquitin ligase. In the current study, RNA sequencing and proteome-wide analysis data indicate that CDC20, a cell cycle regulator, is the E3 ubiquitin ligase most significantly regulated by PRMT6, and the experimental results also verified that PRMT6 promotes the expression of CDC20. However, the finding of Larsen et al. suggest that arginine methylation sites are enriched on CDC20 [28]. Our qRT-PCR and immunoblotting data showed that PRMT6 promotes CDC20 expression through transcriptional regulation, and Co-IP results imply that PRMT6 does not interact with CDC20 (Fig. S5B). PRMT6 can regulate gene transcription through modulating histone methylation, most notably asymmetric di-methylation on arginine 2 of histone 3 (H3R2me2a) [48], and ChIP-qPCR experiment confirmed that PRMT6 promotes transcriptional regulation of CDC20 through H3R2me2a on CDC20 promoter. Therefore, the post-translational modification mechanism is not involved in the regulation of CDC20 expression by PRMT6. Although most previous studies have shown that PRMT6 deposits H3R2me2a at the promoter of target genes to reduce target genes transcription, the work of Bouchard et al. suggests that PRMT6 can also promote the transcriptional activity of downstream genes through H3R2me2a [49]. CDC20 acts as a regulatory protein in a variety of human tumors, interacts with several other proteins at multiple points in the cell cycle, including Cyclin B1, Cyclin A, and CDKN1A [24]. However, it is unknown whether CDC20 can interact with CDKN1B and mediate its expression. In our study, we found that the rescue of CDC20 re-suppresses the expression of CDKN1B in PRMT6-silenced GBM cells, and identified that CDC20 interacts with and destabilizes CDKN1B via its WD40 repeat domain. Notably, a series of rescue assays results demonstrated that PRMT6 contributes to GBM cell proliferation via CDC20-mediated inhibition of CDKN1B expression.

One of the important findings of this study is that EPZ020411 (PRMT6 inhibitor) exhibits a significant anti-GBM effect. Currently, although multiple PRMT6 inhibitors are available, the one with the best specificity and the broadest is EPZ020411 [22, 50]. We have confirmed that the exposure of GBM cells to EPZ020411 leads to significant inhibition of CDC20, H3R2me2a expression, and cell proliferation, while inducing the expression of CDKN1B. Further, the use of EPZ020411 results in the cell cycle of GBM cell arrest in the G0/G1 phase. Our data, therefore, demonstrate that EPZ020411 exhibits a good anti-cancer effect in GBM cells, and also provides a theoretical basis for the clinical treatment of GBM patients.

In conclusion, our findings elucidate that PRMT6 is an epigenetic mediator that promotes CDC20 transcription through histone arginine methylation (H3R2me2a) to mediate ubiquitination and degradation of the cell cycle G1/S phase repressor CDKN1B to facilitate GBM tumorigenesis and progression. Given the feasibility of targeted therapy, we preliminarily demonstrated that a small molecule inhibitor of PRMT6 has a good anti-proliferative effect on GBM cells in vitro. In our study, the PRMT6-CDC20-CDKN1B axis regulates glioma cell cycle progression is identified, therefore PRMT6 and CDC20 functionally play critical roles in GBM. Nonetheless, we should perform further investigations to understand the biological significance of targeting PRMT6 and CDC20 in combination with PRMT6 and CDC20 inhibitors to synergistically attenuate GBM cell proliferation in vitro and in vivo. In addition, PRMT6 could mediate malignant phenotypes such as cell invasion, migration, and chemotherapy resistance in tumors [18], implying that we further explore other roles and mechanisms of PRMT6 in GBM. Despite the limitations of the study, our findings highlight the PRMT6-CDC20 axis as a potentially effective target for therapeutic intervention in GBM.

## Materials and methods

### Clinical specimen and ethical statement

40 human glioma tissues (WHO II-III grade: 24 samples; WHO IV grade: 16 samples) were collected from patients who were initially diagnosed as malignant gliomas at the Department of Neurosurgery, the Second Affiliated Hospital of Guangzhou Medical University (Guangzhou, Guangdong, China). 4 normal brain tissues (NBT) were collected from brain trauma patients as the control group. The Ethical Committee of the Second Affiliated Hospital of Guangzhou Medical University approved the collection of all clinical specimens, and each patient or legal guardian provided written informed permission.

### Cell culture

Human GBM cell lines (U87, U251, T98, SNB19, LN229), Normal Human Astrocytes (NHA), and HEK293T cells were obtained from the Culture Collection of the Chinese Academy of Sciences (Shanghai, China). All of the cells were grown with 10% fetal bovine serum (FBS; Gibco) in DMEM (Gibco, MD, USA), and maintained at 37 °C under 5% CO_2_.

### Plasmids, shRNAs, and siRNAs

The pcDNA3.1-PRMT6-FLAG, pcDNA3.1-CDC20-HA, pcDNA3.1-CDC20(N1-171)-HA, pcDNA3.1-CDC20(N172-499, WD40)-HA, pcDNA3.1-CDKN1B-FLAG, pcDNA3.1-Ubiquitin-His, pLenti-CBH-CDC20-HA overexpression plasmids were constructed from YouBio Biotechnology (Changsha, China). hU6-MCS-Ubiquitin-shPRMT6 lentiviral shRNA plasmids and CDC20 siRNAs were generated by GenePharma (Suzhou, China). The sequences of shPRMT6 and siCDC20 are listed in Table S1. The plasmids or siRNAs were transfected into cells using Lipofectamine 3000 (Invitrogen, USA) according to the manufacturer’s instructions.

### Quantitative Real-Time PCR assay (qRT-PCR)

Total RNA was isolated from cells by TRIZOL reagent (Invitrogen, USA). RNA was reverse transcribed into cDNA by a cDNA synthesis kit (Invitrogen, USA). qRT-PCR was performed using SYBR Green (Takara, Beijing, China) in an ABI 7000 thermal cycler. The 2^−ΔΔCT^ method was used to analyze RNA expression. β-actin was utilized as an internal control for normalization purposes. The primer sequences were synthesized by Sangon Biotechnology (Shanghai, China) and are listed in Table S2.

### Chromatin immunoprecipitation (ChIP) qPCR

The cells were crosslinked with 1% PFA for 10 min at RT, and then 0.125 M glycine was added to stop the crosslinking reaction. After washing with pre-cold PBS, the cells were centrifuged, lysed with buffer containing Protease Inhibitor Cocktail, and centrifuged again to obtain cell nuclear. The cell nuclear was ultra-sonicated for 6 min with 4 s ultra-sonication at 8 s intervals, added elution buffer containing RNase A, and incubated with Proteinase K at 62 °C for 2 h. The fragmented chromatin extract was incubated with antibodies (PRMT6, H3R2me2a, IgG) overnight and then incubated with Protein A/G magnetic beads at 4 °C for 2 h. After thorough washing, elution, and reverse cross-linking, the DNA is purified for qPCR analysis. Production of qPCR reaction was subjected to gel electrophoresis. The primers used for ChIP-qPCR analysis in the promoter are listed in Table S2.

### Protein co-immunoprecipitation assay

For endogenous Co-IP assay, the cell lysate was prepared from U87 or LN229 cells and co-immunoprecipitation was performed using anti-CDC20 antibody (CST, #14866), anti-CDKN1B antibody (CST, #3686), or control IgG as a negative control, followed by immunoblotting analysis with anti-CDC20 and anti-CDKN1B antibodies. For exogenous Co-IP assay, the protein was extracted from HEK 293 T cell with NP-40 lysis buffer. The anti-FLAG antibody (CST, #14793) was fixed on Protein A/G agarose beads (Santa Cruz, sc-2003, USA) and incubated overnight with cell extracts at 4 °C. The beads were analyzed with anti-HA (CST, #3724) and anti-FLAG antibodies.

### RNA sequencing and Tandem Mass Tag (TMT) analysis

Total RNA was isolated using an RNeasy kit (Qiagen) and total protein was extracted with SDT buffer from three independent replicates of the U87 cells with PRMT6 knockdown or control. The RNA amount and purity of each sample were quantified. The protein suspensions were digested with trypsin, and the peptide mixture of each sample was labeled using a TMT reagent according to the manufacturer’s instructions (Thermo Fisher, USA). The work related to LC-MS/MS analysis, library construction, sequencing, and data analysis were carried out by LC-Bio Technology CO., Ltd. (Hangzhou, China). The significance of differentially expressed genes (DEGs) with log2(fold change) > 1 and *P* < 0.05 was determined. The raw data of RNA sequencing was uploaded to GEO and the accession number is GSE221971.

### Statistical analysis

The data were statistically analyzed with SPSS 21.0 (SPSS, Chicago, USA) and GraphPad Prism 8.0. Bars and error represent the mean ± standard deviation (mean ± SD) of at least three independent replicate measurements. The means of normally distributed continuous data between two groups were analyzed by unpaired Student *t* tests. The one-way ANOVA was used to compare the expression level of PRMT6 or CDC20 in patients with different grade glioma. Survival curves were drawn by Kaplan-Meier (KM) and compared by log-rank test. The Pearson analysis method was applied to the correlation between PRMT6 and CDC20 expression in patients with GBM. Statistical significance was defined as *P* < 0.05.

## Supplementary information

supplementary information

## Data Availability

All data created and analyzed during this current work are involved in this published article (and its supplementary information files).
