# Supplementary material for: PRMT6-CDC20 facilitates glioblastoma progression via the degradation of CDKN1B
Source: Oncogene. 2023 Feb 15;42(14):1088–100. doi: 10.1038/s41388-023-02624-7 (PMC10063447; doi:10.1038/s41388-023-02624-7)
Supplement: Supplementary file 1 — supplementary information [file 41388_2023_2624_MOESM1_ESM.docx]

**Supplementary information**

**Supplementary methods**

**Protein half-life assay**

U87, LN229, and T98 cells were treated with an indicated condition and subjected to 100 µg/ml cycloheximide (CHX, Selleck, USA). At 0, 2, 4, 6, 8, and 12 h post-exposure, the cell lysate was prepared, and the results were analyzed by western blotting.

**Lentivirus packaging and generation of stable cells**

Lentivirus packaging was performed in accordance with the method previously described [1]. HEK293T cells were co-transfected with an shPRMT6 plasmid or pLenti-CBH-CDC20-HA overexpression plasmid, Pcmvdr8.91 plasmid, and pMD.G-VSV-G plasmid. The cell supernatants were collected and filtered with a 0.45 µm nitrocellulose filter 48 h post-transfection. Finally, the glioma cells were incubated with the supernatants for 48 h and screened with 5 µg/ml puromycin for 1-2 weeks to obtain stable cells.

**Cell viability assay**

The Cell Counting Kit-8 (CCK-8) test was performed to assess cell viability using a CCK-8 kit (Dojindo, Japan). Stably transfected cells (2 x10^3^ cells/well) were grown in 96-well plates for 0-5 days, respectively. 10% CCK-8 solution was added to the cells and incubated at 37°C for 1 h. The plates were read on a microplate reader (TECAN, Switzerland) at 450 nm wavelength. The colony formation assay was also used to evaluate cell proliferation. The cells (1 x 10^3^ cells/well) were cultured in 6-well plates for 10 days. The colonies were fixed with 4% paraformaldehyde, stained with 0.1% crystal violet, pictured with ChemiDoc™ XRS+ system (Bio-Rad, USA), and counted with Image J software.

**Cell cycle analysis**

A cell cycle assay was performed to analyze cell cycle phase distribution with a kit (Beyotime, Shanghai, China). The cells were harvested and fixed with pre-cold 70% ethanol at 4 °C overnight. Then, the cells were incubated in a 500 µL staining buffer containing 25 µL Propidium Iodide (PI) and 10 µL RNase A at RT for 30 min in the dark. The stained cells were then immediately analyzed with a CytExpert flow cytometer (Beckman Coulter, USA).

**Immunoblot**

RIPA lysis buffer supplemented with protease inhibitors (Beyotime, Shanghai, China) was used to separate total protein from cells and tissues. The concentration of total protein was determined with a BCA kit (Invitrogen, CA, USA). An equal amount of total protein (30 µg) was electrophoresed on 10% -12% SDS-PAGE, and then electro-transferred to a nitrocellulose filter (NC) membrane (Millipore, MA, USA). Then, the membranes were blocked with 5% non-fat dry milk in TBST at RT for 1 h. After washing with TBST, the membranes were incubated with subsequent primary antibodies at 4 °C overnight. On the second day, the membranes were incubated with HRP-labeled secondary antibodies (Jackson, Lancaster, USA) at RT for 1 h. An enhanced chemiluminescence system (Millipore, MA, USA) was used to measure protein expression value. The relative quantity of proteins was analyzed by Image J software. Primary antibodies used for immunoblot were: anti-PRMT6 (CST, #14641, 1:1000), anti-CDC20 (CST, #14866, 1:1000), anti-CDKN1B (CST, #3686, 1:1000), anti-H3R2me2a (Abcam, ab176845, 1:500), anti-Histone H3 (Abcam, ab1791, 1:2000), anti-FLAG (CST, #14793, 1:1000), anti-HA (CST, #3724, 1:1000), anti-CDK6 (CST, #13331, 1:1000), anti-Cyclin D1 (CST, #55506, 1:1000), anti-CDKN1A (CST, #2947, 1:1000), and anti-β-Actin (ABclonal, #AC026, 1:50000).

**Ubiquitylation of CDKN1B *in vivo* and *in vitro***

The control, NC, shPRMT6 U87 cells were transfected with His-ubiquitin plasmid for 48 h, and then the cells were treated with 20 µM MG132 (MCE, USA) for 6 h. The cells were lysed using NP-40 lysis buffer, and the cell lysates were immunoprecipitated using anti-CDKN1B antibody and Protein A/G agarose beads. The beads were washed with NP-40 buffer and analyzed with anti-His (CST, #12698) antibody. HEK293T cells were co-transfected with CDKN1B-FLAG, His-Ubiquitin, and CDC20-HA or siCDC20 for 48h, and then the cells were treated with 20 µM MG132 for 6 h. CDKN1B protein containing ubiquitylated CDKN1B was immunoprecipitated from the cell lysates using FLAG–beads and washed extensively using NP-40 lysis buffer. The result reactions were subjected to immunoblotting analysis.

**Immunofluorescence (IF)**

U87 and LN229 cells were cultured on 35 mm sterile coverslips with applicable density for 24 h. Then, the cells were washed with pre-cold PBS and fixed with 4% PFA for 30 min. The cells were then incubated with 0.5 % Triton X-100 for 10 min at RT. After washing with PBS, the cells were blocked with 10% BSA at RT for 1 h before being incubated with target primary antibodies overnight at 4 °C. On the second day, the cells were incubated with fluorescent secondary antibodies (Alexa Fluor® 488 or Alexa Fluor® 555) at RT for 1 h in the dark. Finally, the cell nucleus was stained with DAPI, and images of immunofluorescence staining were captured by a fluorescence microscope. Primary antibodies used for IF were: anti-CDC20 (Proteintech, 10252-1-AP, 1:400) and anti-CDKN1B (Santa Cruz, sc-1641, 1:50).

**Immunohistochemistry (IHC)**

The clinical glioma tissues and brains of xenograft mice were fixed with 4% PFA and embedded in paraffin. Then, 5 µm slices were cut by a microtome (Leica, Germany) and deparaffinized, dehydrated, and incubated in heat-mediated antigen retrieval. Subsequently, the endogenous catalase was eliminated with 3% H_2_O_2_‐methanol, and tissue slices were incubated with indicated primary antibodies at 4 °C overnight. On the next day, sections were washed with PBS and incubated with biotinylated secondary antibodies at RT for 1 h. They were then incubated with peroxidase solution for 30 min and then the sections were stained with DAB reagent and counterstained with hematoxylin. The images of each section were taken and analyzed under an optical microscope. IHC staining images were quantified by ImageJ software. Primary antibodies used for IHC were: anti-PRMT6 (Proteintech, 15395-1-AP, 1:200), anti-CDC20 (Proteintech, 10252-1-AP, 1:150), anti-CDKN1B (Santa Cruz, sc-1641, 1:100), and anti-Ki67 (Abcam, ab15580, 1:500).

**Xenograft tumor assay**

All mouse experiments were approved by the Institutional Animal Care and Use Committee of Guangzhou Medical University. U87 cells (5×10^5^ in 10 µL PBS) were injected intracranially into female 6-week-old BALB/c nude mice (Guangdong Medical Laboratory Animal Center, Guangzhou, China) (5 mice for each group). Mice were observed daily for death or neurological symptoms and then sacrificed if they developed neurological symptoms. The entire brain was then collected, fixed with 4% PFA, embedded in paraffin, and sectioned coronally from anterior to posterior. To measure the size of the tumor, the largest cross-section area of the tumor was selected. The formula for calculating the tumor volume is V = (a × b^2^) / 2, where a is the longest diameter and b is the shortest diameter. a and b are measured with Image J. For survival analysis assays, mice (6 mice for each group) were injected intracranially with the above procedure. The dying mice were sacrificed under deep anesthesia and the remaining mice were sacrificed 90 days after injection of U87 cells.

**Bioinformatics analysis**

PRMT6 or CDC20 expression and survival time data of patients with glioma/GBM from the TCGA, CGGA, and Gravendeel databases were analyzed in this study by the GlioVis website (http://gliovis.bioinfo.cnio.es/). The samples of survival analysis were divided into high and low groups followed by PRMT6 or CDC20 expression values. Gene functional enrichment analysis was performed by the Metascape website (https://metascape.org). Graphic production and statistical analysis were performed by GraphPad Prism 8.0 (GraphPad Software, La Jolla, CA, USA).

**REFERENCES**

1. Yu S, Yu X, Sun L, Zheng Y, Chen L, Xu H, et al. GBP2 enhances glioblastoma invasion through Stat3/fibronectin pathway. Oncogene. 2020;39:5042-5055.

**Supplementary figures**


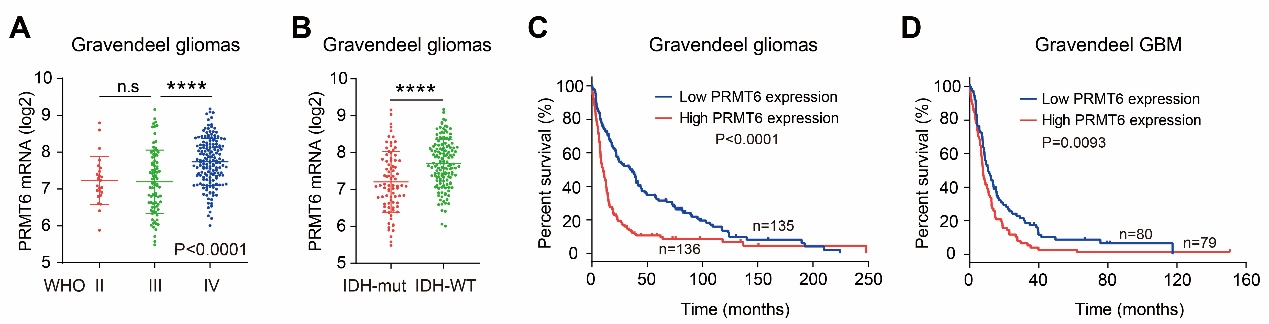


**Fig. S1 PRMT6 is overexpressed in glioma. A**, **B** PRMT6 mRNA expression in gliomas with different WHO grades and different IDH statuses from the Gravendeel database. **C**, **D** KM survival curves of patients with glioma and GBM from the Gravendeel database stratified by PRMT6 expression. n.s: no significant, *****p* < 0.0001.

**
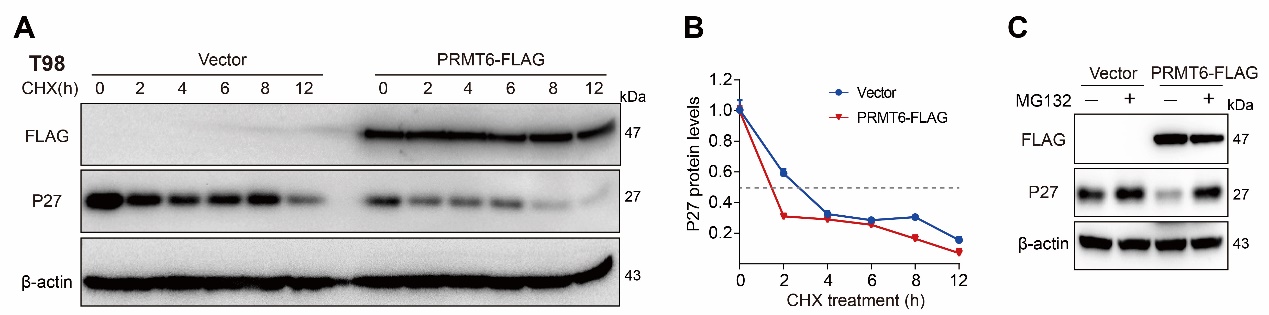
**

**Fig. S2 PRMT6 attenuates the protein stability of CDKN1B in T98 cells. A** Immunoblotting analysis of T98 cells with or without PRMT6 overexpression to determine the protein half-life of CDKN1B. **B** Quantifications of the protein half-life result. **C** T98 cells, with or without PRMT6 overexpression, were treated with vehicle control or with MG132 (20 μM) for 6 h, and the abundance of CDKN1B was determined by western blotting.


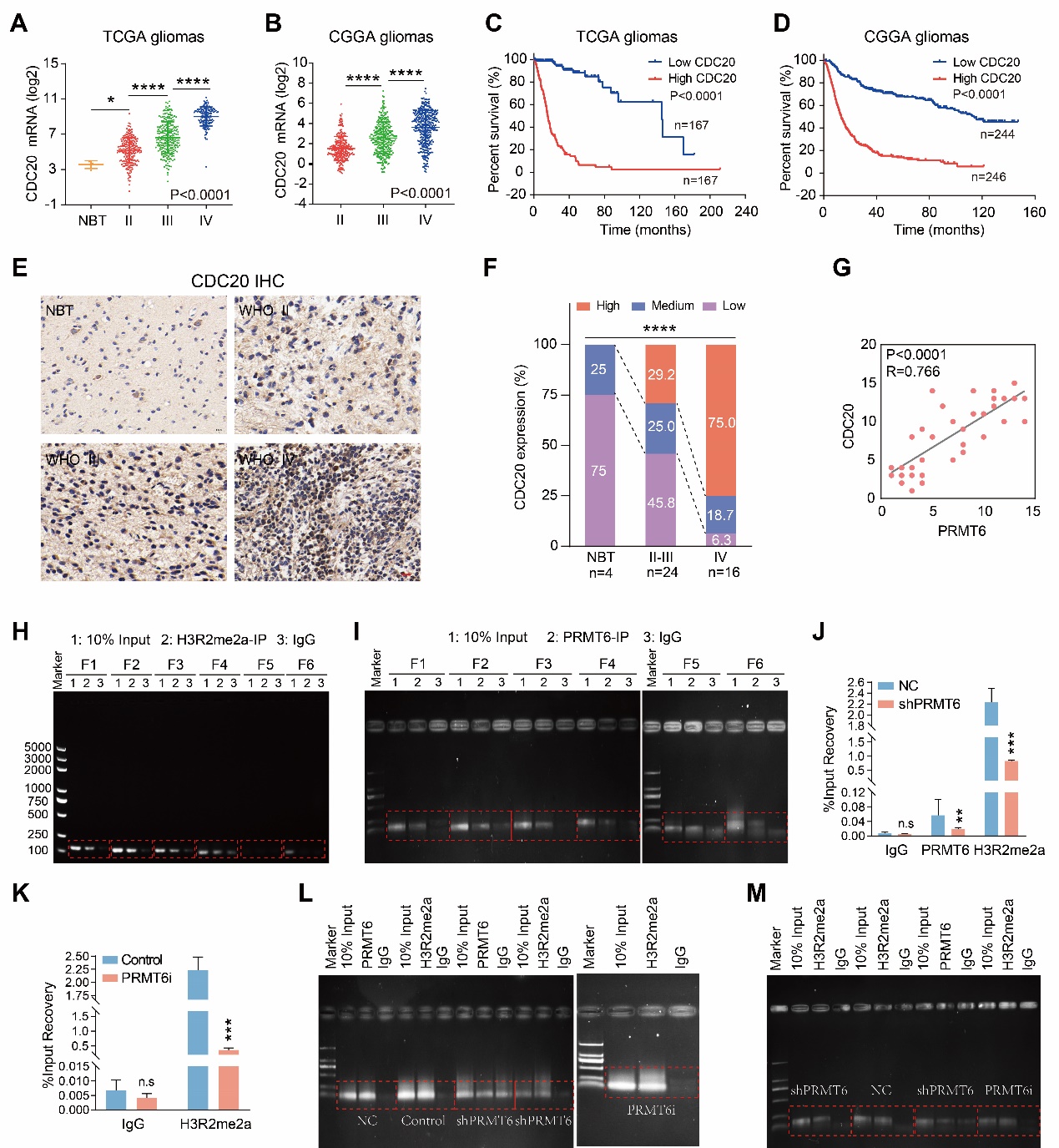


**Fig. S3 CDC20 is overexpressed in glioma. A**, **B** CDC20 mRNA expression in gliomas with different WHO grades from TCGA and CGGA databases. **C**, **D** Kaplan-Meier survival curves of patients with glioma from TCGA and CGGA databases stratified by CDC20 expression. **E** Representative IHC images of CDC20 in gliomas with different histological grades and NBT. Bar: 20 µm. **F** The semi‑quantitative for the IHC results of PRMT6 and CDC20. **G** Pearson correlation analysis of IHC score statistics for PRMT6 and CDC20. **H, I** The ChIP-qPCR products of PRMT6 and H3R2me2a in the promoter region of CDC20 were analyzed on agarose gel electrophoresis. **J, K** ChIP-qPCR was performed to determine H3R2me2a in the promoter of CDC20 (F4) in U87 cells with or without PRMT6 knockdown (shPRMT6) and U87 cells with or without PRMT6 inhibition (PRMT6i). **L, M** The ChIP-qPCR products of PRMT6 and H3R2me2a in the promoter region of CDC20 (F3 and F4) in U87 cells with or without PRMT6 knockdown (shPRMT6) and U87 cells with or without PRMT6 inhibition (PRMT6i) were analyzed on agarose gel electrophoresis. n.s: no significant, **p* < 0.05, ***p* < 0.01, ****p* < 0.001, *****p* < 0.0001.


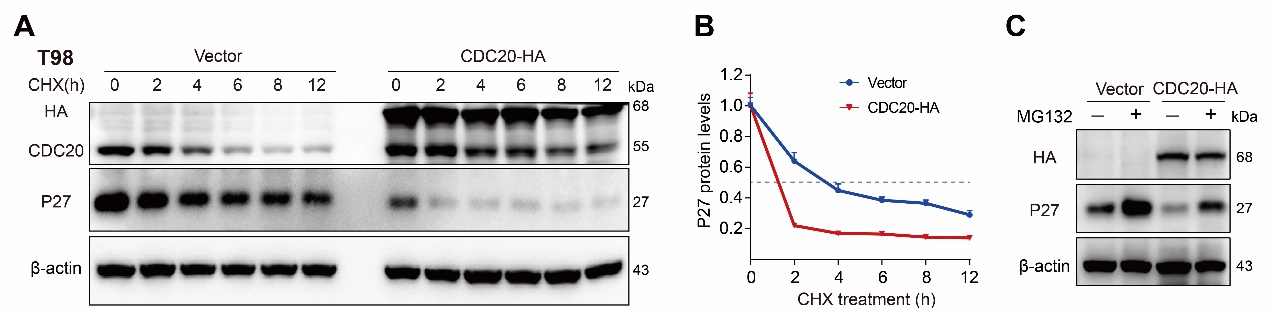


**Fig. S4 CDC20 attenuates the protein stability of CDKN1B in T98 cells. A** Immunoblotting analysis of T98 cells with or without CDC20 overexpression to determine the protein half-life of CDKN1B. **B** Quantifications of the protein half-life result. **C** T98 cells, with or without CDC20 overexpression, were treated with vehicle control or with MG132 (20 μM) for 6 h, and the abundance of CDKN1B was determined by western blotting.


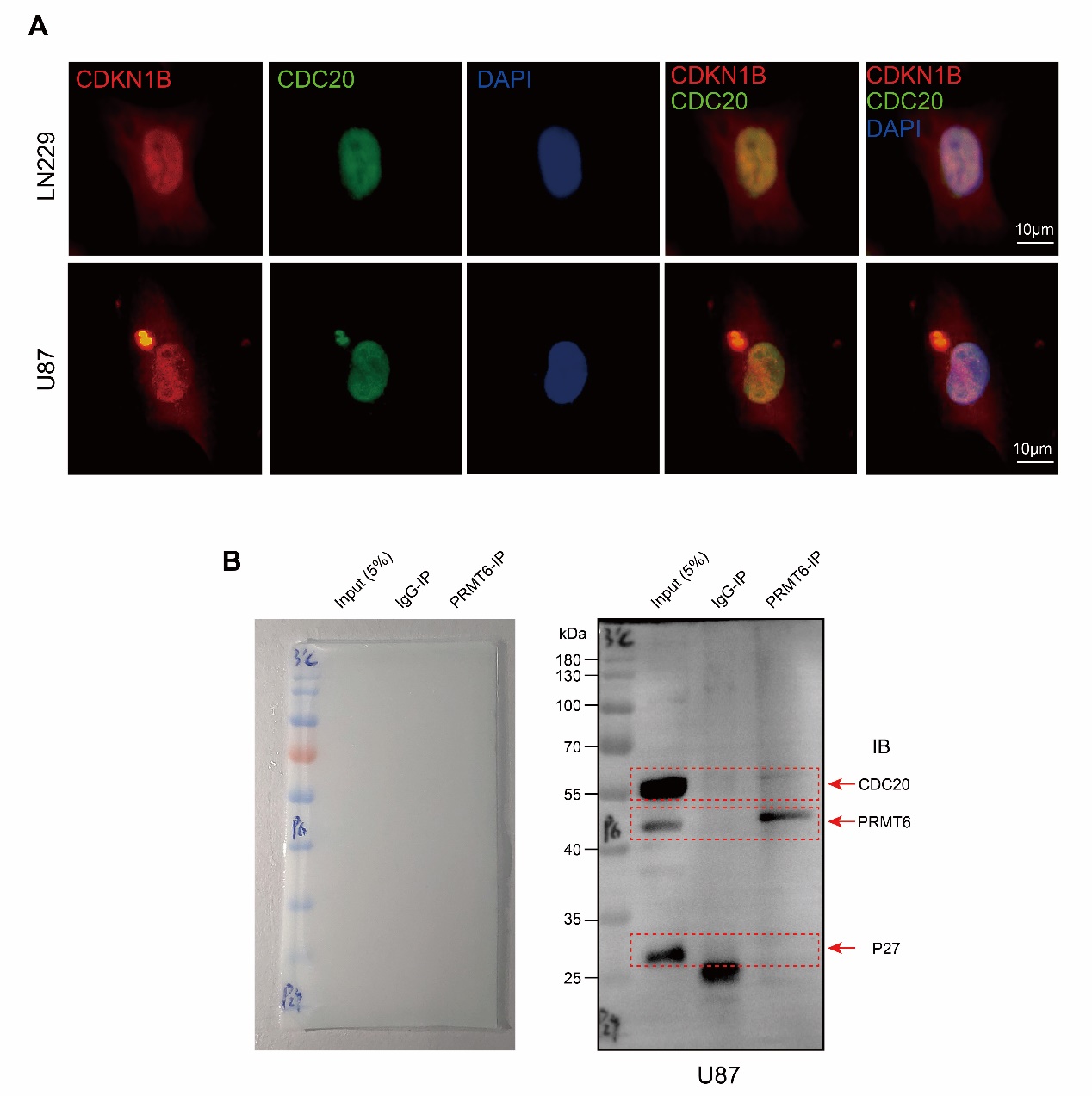


**Fig. S5 CDC20 interacts with CDKN1B. A** CDKN1B (red), CDC20 (green) and DAPI (blue) expression in LN229 and U87 cells were visualized under fluorescence microscope. **B** The interaction of endogenous PRMT6 with CDC20 or CDKN1B was evaluated by Co-IP assay in U87 cells.

**
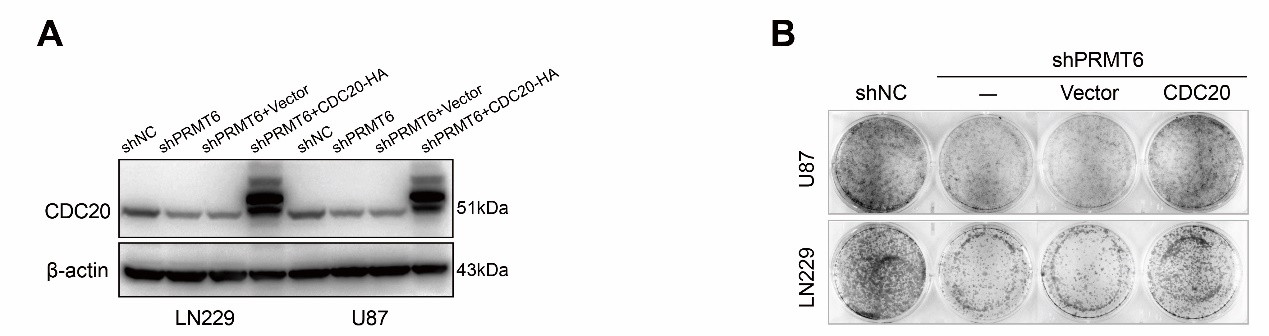
**

**Fig. S6 PRMT6 promotes GBM cell colony formation via CDC20 *in vitro*. A** Immunoblot analysis of CDC20 expression in U87 and LN229 cells treated with NC shRNA, shPRMT6, shPRMT6 + CDC20 Vector or shPRMT6 + CDC20. **B** Colony formation capability for U87 and LN229 cells infected with NC shRNA, shPRMT6, shPRMT6 + CDC20 Vector or shPRMT6 + CDC20.

**Supplementary tables**

**Table S1. Sequences of siRNAs and shRNAs.**

| **shRNA/siRNA** | **Sequence (5'-3')** |
| --- | --- |
| shRNA_NC | CCGGCAACAAGAT GAAGAGCACAACTCGAGTTGGTGCTCTTCATCTTGTTGTTTTT |
| shPRMT6 #1 | CCGGGCCCAGTTTGAGATGCCTTATCTCGAGATAAGGCATCTCAAACTGGGCTTTTTG |
| shPRMT6 #2 | CCGGCACCGGCATTCTGAGCATCTTCTCGAGAAGATGCTCAGAATGCCGGTGTTTTTG |
| shPRMT6 #3 | CCGGCACGGACGTTTCAGGAGAGATCTCGAGATCTCTCCTGAAACGTCCGTGTTTTTG |
| siRNA_NC | UUCUCCGAACGUGUCACGUTT |
| siCDC20 #1 | CGAAAUGACUAUUACCUGAAC |
| siCDC20 #2 | GGAUGUGCAGCAGCAGAAACG |

**Table S2. Primer sequences for RT-qPCR and ChIP-qPCR.**

| **Genes** | **Sequence (5'-3')** |
| --- | --- |
| CDK6 | F: TCTTCATTCACACCGAGTAGTGC |
|  | R: TGAGGTTAGAGCCATCTGGAAA |
| Cyclin D1 | F: TGGAGCCCGTGAAAAAGAGC |
|  | R: TCTCCTTCATCTTAGAGGCCAC |
| P21 | F: CGATGGAACTTCGACTTTGTCA |
|  | R: GCACAAGGGTACAAGACAGTG |
| P27 | F: ATCACAAACCCCTAGAGGGCA |
|  | R: GGGTCTGTAGTAGAACTCGGG |
| CDC20 | F: GCACAGTTCGCGTTCGAGA |
|  | R: CTGGATTTGCCAGGAGTTCGG |
| β-actin | F: TCCTGTGGCATCCACGAAACT |
|  | R: GAAGCATTTGCGGTGGACGAT |
| CDC20 F1 | F: TGATTGCTGCTCCACCTCTG |
|  | R: GGATGTGCCCGAGGTCATAC |
| CDC20 F2 | F: TGGATTTGTTGTCGGGGTGG |
|  | R: GCACTGTGGCTTATCCCCTT |
| CDC20 F3 | F: AGCAAACGAGACAAACACACG |
|  | R: GAGCAGGAAGAAGGCCAGAG |
| CDC20 F4 | F: TTCTGCACCGAGTTCTGCAT |
|  | R: TCCTGGAGTCGTCTCGGTTC |
| CDC20 F5 | F: GGCCGGCTTTCCAGTACTAG |
|  | R: TCCGGGGAAAGTCTCAGCTA |
| CDC20 F6 | F: GCGGAGAGTTTAAGAGGCGT |
|  | R: GCCACAAAATCAGGGCCAC |
